# Ruptured ovarian ectopic pregnancy in a primigravid woman: A case report

**DOI:** 10.18502/ijrm.v22i2.15713

**Published:** 2024-03-25

**Authors:** Fahimeh Sadat Tabatabaei Mirokabad, Mohammad Poorebrahimi, Sajad Zare Garizi, Razieh Sadat Tabatabaei

**Affiliations:** ^1^Department of Gynecology and Obstetrics, Shahid Sadoughi University of Medical Sciences, Yazd, Iran.; ^2^Student Research Committee, Shahid Sadoughi University of Medical Sciences, Yazd, Iran.; ^3^Mother and Newborn Health Research Center, Shahid Sadoughi University of Medical Sciences, Yazd, Iran.

**Keywords:** Ectopic pregnancy, Pregnancy, Ovarian, Laparotomy.

## Abstract

**Background:**

Ovarian ectopic pregnancy (EP) is one of the rare forms of EP. The use of intrauterine devices and assisted reproduction techniques are among the most important risk factors for ovarian EP. Clinical signs are usually menopause, abdominal pain, and vaginal bleeding. Definitive diagnosis of ovarian EP before its rupture remains a serious challenge and, in most cases, it is diagnosed after rupture when medical treatment has no place and surgery becomes necessary.

**Case Presentation:**

Here, we report a 35-yr-old primigravida woman referred to Shahid Sadoughi hospital, Yazd, Iran with abdominal pain and sudden loss of consciousness. An initial evaluation was done and she underwent laparotomy.

**Conclusion:**

The preferred treatment for ovarian EP is to surgically remove the gestational sac and preserve as much ovarian tissue as possible. However, some cases, like ours, need a complete or partial oophorectomy.

## 1. Introduction

Ectopic pregnancy (EP) is one of the most important causes of maternal morbidity and mortality, which is reported in 0.5–2% of pregnancies (1). Ovarian EP is a rare and fatal type of EP, accounting for approximately 0.5–3% of all ectopic pregnancies (2). Studies showed an increase in the prevalence of ovarian EP (3). Ovarian EP can be classified into intrafollicular (primary) and extrafollicular (secondary). In the intrafollicular the egg is fertilized before leaving the ovary while it is inside the follicle and in the extrafollicular, fertilization occurs when the egg in the fallopian tube is released and replaced in the ovarian stroma (4, 5). Due to the ovarian cortex and its lack of elasticity, in most cases ovarian EP has to be ruptured during the first trimester, of which 5.3% reach the second and only 3.7% reach the third trimester (6). Although the gestational sac is usually intact, the fallopian tubes remain normal, and hemorrhagic lesions may appear on the ovary with or without fetal tissue in the pelvic cavity (7). On the other hand, premature rupture can lead to hemoperitoneum and hypotension which is considered as one of the life-threatening gynecological emergencies (4).

Here, we report a case of a primigravida woman with a previous diagnosis of intrauterine pregnancy with sudden loss of consciousness, and abdominal pain who underwent laparotomy.

## 2. Case Presentation

**Figure 1:**
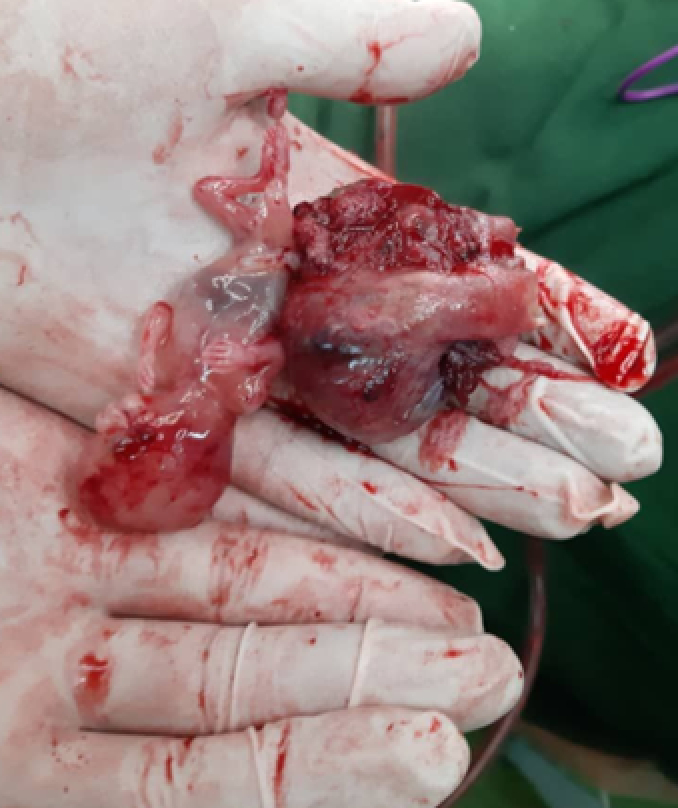
An 11-wk fetus and the ruptured ovary after surgical resection.

**Figure 2:**
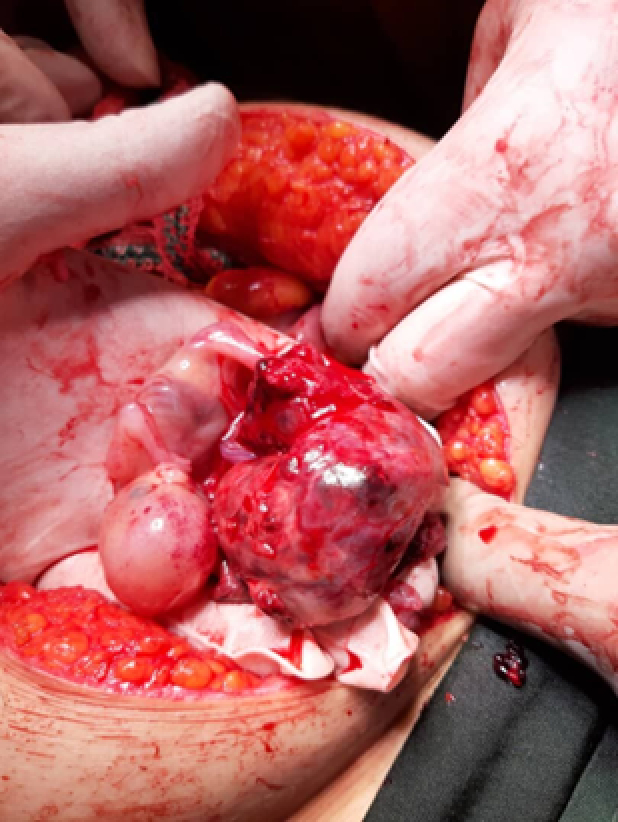
Fetus tissue and ruptured ovary during laparotomy.

A 35-yr-old primigravida woman with a previous diagnosis of intrauterine pregnancy was admitted to the Shahid Sadoughi hospital, Yazd, Iran at the 11 ${\;}^{\text{th}}$ wk after last menstrual period, with sudden loss of consciousness. An intrauterine fetus was reported a few hours after performing a nuchal translucency scan. Despite a 10-yr history of infertility and irregular menstruation, she had not been treated with assisted reproductive procedures. Vital signs were stable at first, and there was only mild abdominal tenderness. She underwent a focused assessment with sonography in trauma examination, and mild free fluid was reported in the pelvic cavity. After 2 hr, she developed acute abdominal pain and had a severe drop in blood pressure (systolic blood pressure: 80, diastolic blood pressure: 50), which indicates emergency laparotomy due to hemodynamic instability and the lack of a definitive diagnosis. During laparotomy, about 2000 cc of blood inside the abdominal cavity and a ruptured ovarian EP were observed (Figures 1 and 2). The uterus was observed with a normal size, the blood inside the uterus was removed by suction. Salpingo-oophorectomy was performed and the histopathology report confirmed the EP.

### Ethical considerations

A written informed consent was obtained to use her medical records for any research purpose.

## 3. Discussion

Although ovarian EP is a very rare condition; however it is on the verge of getting common. Ovarian EP occurs by either secondary implantation or follicular extrusion disorder (2). Studies show that all known risk factors in tubal pregnancy, such as pelvic cavity inflammation or fallopian tube epithelial damage, are not very important in ovarian EP. The main risk factors for ovarian EP include the use of intrauterine devices, assisted reproductive techniques, concurrent endometriosis, and the history of intrauterine surgeries (8, 9). The most common clinical symptom of ovarian EP is abdominal pain with vaginal bleeding or menstrual disorders, and in case of occurring these symptoms with a history of previous amenorrhea, a high beta-human chorionic gonadotropin level, and absence of gestational sac in the uterine cavity on ultrasound, an ovarian EP should be evaluated (10). The most important ultrasound finding in ovarian EP is different echogenic from the ovarian tissue, which is placed on the ovary, and even parts of the fetus are visible in some cases (11). One of the most important challenges in ovarian EP diagnosis is to differentiate it from ruptured corpus luteum, hemorrhagic ovarian cyst, or tubal abortion both in ultrasound or surgery. The report of the decreased wall echogenicity compared with the endometrium and an anechoic texture in ultrasound suggests a corpus luteum, but it is not certain (12). Color Doppler imaging is not useful in distinguishing ovarian EP from other types of EP and tubal pregnancy (13). Therefore, ultrasound diagnosis of ovarian EP is difficult and it is usually diagnosed during surgery (14). The diagnosis can be confirmed only by histologic examination if fetal parts cannot be seen macroscopically during the operation (15).

## 4. Conclusion

Ovarian EP is one of the rare and dangerous types of EP, which is increasing due to an increase in infertility and the use of assisted reproductive techniques. Diagnosis of ovarian EP and its management is considered a serious challenge. Ultrasound plays a significant role in the diagnosis of ovarian EP in unruptured cases, but most women present with a ruptured EP. So, ultrasound imaging cannot be helpful in definitive diagnosis. Surgery is the gold standard for ovarian EP diagnosis and treatment, and ovarian resection is also required in many cases.

##  Data availability

The data that support the findings of this study are available from the corresponding author upon reasonable request.

##  Author contributions

P.M. and ZG.S. designed the study. TM.FS. and T.RS. identified and collected the data. P.M., TM.FS. and ZG.S. wrote and reviewed the paper. T.RS. critically reviewed the paper. All authors approved the final version of the paper and took responsibility for its content.

##  Conflict of Interest

The authors declare that there is no conflict of interest.
